# Awareness of FAIR and FAIR4RS among international research software funders

**DOI:** 10.1038/s41597-025-04820-4

**Published:** 2025-04-15

**Authors:** Eric A. Jensen, Daniel S. Katz

**Affiliations:** 1 University of Illinois Urbana-Champaign, Urbana, USA; 2 Department of Sociology, University of Warwick, Coventry, United Kingdom; 3 Research Department, Institute for Methods Innovation, Dublin, Ireland

**Keywords:** Funding, Policy, Research data, Institutions

## Abstract

Research software has become indispensable in contemporary research and is now viewed as essential infrastructure in many scholarly fields. Encompassing source code, algorithms, scripts, computational workflows, and executables generated during or specifically for research, it plays a critical role in advancing scholarly knowledge. The research software field includes considerable open-source use and links to the broader open science movement. In this context, it has been argued that the well-established FAIR (Findable, Accessible, Interoperable, Reusable) principles for research data should be adapted for research software under the label FAIR4RS. However, the level of uptake of FAIR4RS principles is unclear. To gauge FAIR4RS’s status, international research funders involved in supporting research software (n = 36) were surveyed about their awareness of the concept. The survey reveals much greater familiarity with the more established FAIR principles for data (73% ‘extremely familiar’) than FAIR4RS (33% ‘extremely familiar’). Nevertheless, there is still considerable recognition of the relatively new FAIR4RS concept, a significant achievement for efforts to extend open science policies and practices to encompass research software.

## Introduction

The FAIR principles—Findable, Accessible, Interoperable, and Reusable—are a cornerstone for aligning data management and sharing with good practices in research and scholarship. These guidelines are designed to enhance the utility and impact of scientific data by ensuring that datasets are easily located, as accessible as possible under clear conditions, compatible with other datasets, and structured to enable reuse^1^. The FAIR framework fosters transparency, reproducibility, and innovation across research disciplines. At this point, international research funders and academics have widely recognized the FAIR principles and taken steps to advance its implementation^2,3^. Chue Hong *et al*.^4^ present a community-developed adaptation of the original FAIR principles specifically for research software (i.e., FAIR4RS)^5^. This development was based on the fact that while the FAIR principles were initially developed for data, “research software is now being understood as a type of digital object to which FAIR should be applied”^5^. Application of FAIR principles to research software is essential due to the unique challenges associated with software, such as its executability, continuous evolution, and versioning. This adaptation reflects the growing recognition within the research community of software’s critical role in advancing scholarly discovery. Like FAIR for data before it, the FAIR4RS principles aim to ensure that research software is discoverable, reusable, and sustainable, thus enhancing transparency and reproducibility in research. Barker *et al*.^5^ argue that “the development of the FAIR4RS Principles is a milestone for the research community in recognizing the increasing value of research software as fundamental and vital to research worldwide.”

Research software has become indispensable in contemporary research and is now viewed as essential infrastructure in many scholarly fields^5–9^. Encompassing source code, algorithms, scripts, computational workflows, and executables generated during or specifically for research, it plays a critical role in advancing scholarly knowledge. As computational methods increasingly take center stage across research disciplines, the importance of developing, maintaining, and maximizing the impact of research software has never been more apparent^10^. Recognizing this, some research funders have started creating programs and policies to support and enhance the role of research software^11,12^. Research software funding comes from government and philanthropic sources^13,14^. Often, this software is open source^11,12^ aligning itself with the broader open science movement and good practices such as the FAIR data principles^15^.

To date, there has been no empirical study of how international research funders understand and apply the FAIR and FAIR4RS principles to their programs^16^. Given the critical role of research funders within the scholarly ecosystem, the extent to which these fundamental concepts have become integrated into funders’ thinking is a crucial indicator of the evolution of this field of practice. Here, we draw on part of the dataset from a survey of international research funder representatives to address the following research question: To what extent are research funding organizations that support research software aware of FAIR4RS? How does awareness of FAIR4RS compare to a baseline of awareness of FAIR principles among these research software funders?

**Fig. 1:**
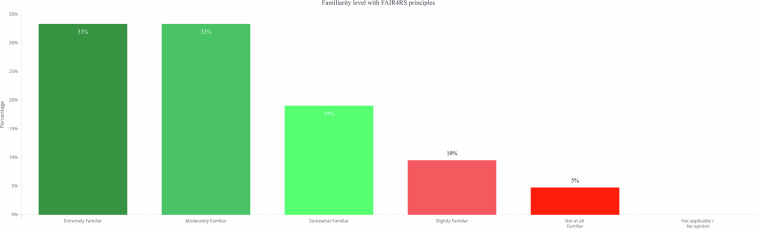
Research software funders’ familiarity with FAIR4RS principles.

## Methods

This research employed a mixed methods online survey to investigate research software funders’ perspectives.

All participants gave informed consent at the start of the online survey. The University of Illinois Urbana-Champaign Institutional Review Board (no. 24374) reviewed the study and determined it exempt.

Data collection took place from December 2023 to May 2024. The mean completion time for the detailed survey was 28 minutes and 13 seconds. The data were cleaned and prepared for analysis by removing any identifiable respondent details. The dataset underpinning this article has been published on Zenodo, along with the full survey instrument used for the study^17^. All participants provided explicit informed consent to participate in this study at the start of the online form used to gather the survey data. This included consent for data to be used for research purposes and to be published as open data after deidentification.

### Survey design

The survey began by collecting profile information, including institutional affiliation and job title. The survey primarily gathered detailed information about initiatives, policies, or programs to support research software but also included a much smaller set of questions about additional topics, such as strategic funding priorities and awareness of key concepts. The data generated from this survey are too extensive to report in a single manuscript. Here, we focus on the results generated via the set of questions asking about FAIR and FAIR4RS, specifically, the following survey items shown in Table 1.

**Table 1:** Survey items to measure awareness of FAIR and FAIR4RS.

Variable	Survey item	Response options
Awareness of FAIR principles	“Have you ever heard of the FAIR (findable, accessible, interoperable, and reusable) principles for data?”	Yes, No, Unsure (If ‘Yes’, then the next question was asked)
	“How familiar are you with the FAIR principles for data?”	Not at all Familiar, Slightly Familiar, Somewhat Familiar, Moderately Familiar, Extremely Familiar
Awareness of FAIR4RS principles	“Have you ever heard of the FAIR4RS principles for research software?”	Yes, No, Unsure (If ‘Yes’, then the next question was asked)
	“How familiar are you with the FAIR4RS principles for research software?”	Not at all Familiar, Slightly Familiar, Somewhat Familiar, Moderately Familiar, Extremely Familiar

In addition, an open-ended question asked for further details about the respondents’ assessments of FAIR4RS’s relevance to their work.

### Sampling

The survey targeted international research funders, including governmental and non-governmental (e.g., philanthropic) organizations. An initial contact list was created based on participation in the Research Software Association (ReSA) and known responsibilities for research software funding among the authors’ networks. This list was refined by removing individuals who had moved to unrelated professional roles or were unavailable long-term due to personal issues.

The final contact list comprised 71 people at 37 funding organizations. After excluding individuals when a member of their organization had already provided a complete response or when the person was no longer working on a relevant topic or was otherwise unavailable (total of n = 30), 41 people remained. Of these, five did not complete the survey, while 36 individuals (representing 30 research funding organizations) did, yielding a response rate of 87.8% (and representing 81% of the original organizations). Fully completed survey responses were not required for inclusion in the sample, resulting in varied sample sizes across different survey questions.

The respondents represented governmental (79%; n = 26), philanthropic (18%; n = 6), and corporate (3%; n = 1) research funders. No published ground truth exists on the global population of research funders in general or on research software funders, specifically. This makes it difficult to assess this sample’s representativeness. The 2024 International Research Software Funders Forum event targeted precisely the population of international research software funders. In this event, the participating funding organizations were 68% governmental (n = 17) and 32% philanthropic (n = 8), with no corporate funders represented^18^. This suggests that our sample may be modestly over-representing governmental research software funders. However, it is also possible that governmental funding organizations would be slightly under-represented at an in-person event.

Respondents’ job titles spanned the following categories: Senior Leadership and Executive (e.g., Vice President of Strategy); Program and Project Management (e.g., Senior Program Manager); Planning and Business Development; and Scientific, Technical, and IT roles (e.g., Scientific Information Lead).

Most respondents, 72.7% (n = 24), answered “Yes” to the question, “Has your organization established any policies, initiatives, or programs aimed at supporting research software?” Meanwhile, 18.2% (n = 6) said “No,” and 9.1% (n = 3) were “Unsure.”

Regarding geographic distribution in the achieved sample, most survey respondents were from North America and Europe, with 15 (43%) and 12 (34%) participants, respectively. The sample also comprised 4 (11%) participants from South America, 3 (9%) from Oceania, and 1 (3%) from Asia, reflecting a global but uneven representation across continents. This unevenness does not necessarily signify a lack of representativeness since the population of research funders that have a focus on research software is likely to be geographically skewed towards North America and Europe at present. This can also be inferred from the 2024 International Research Software Funders Forum event participation profile: Participating organizations were concentrated in Europe (48%; n = 12) and North America (44%; n = 11), with limited representation from Oceania (4%; n = 1) and Asia (4%; n = 1), and no participating funders from South America^18^. This comparison suggests that our survey sample may be slightly more geographically diverse than the target population of research software funders.

Some participating funders covered a broad spectrum of disciplines, while others focused on specific domains such as social sciences, health, environment, physical sciences, or humanities.

## Results

### Awareness of FAIR data principles

The survey revealed a remarkably high level of awareness of FAIR principles for data, with 97% (n = 30) answering ‘yes’ to the question about whether they had heard of FAIR for data and just 3% (n = 1) saying no.

There was more differentiation in the follow-up question about familiarity with the FAIR principles for data that was asked only of those who said ‘yes’ (that they had heard of FAIR for data) (Fig. 1). Yet, the responses were still overwhelmingly positive, with 73% self-reporting that they were “extremely familiar” with FAIR data principles (n = 19). The remaining 27% (n = 7) said they were “moderately familiar,” the next highest level of familiarity available as a response option.

### Awareness of FAIR4RS principles

Results show substantial awareness of FAIR4RS but lagging the FAIR for data baseline.

Among the survey respondents for this question, 70% indicated that they had heard of the FAIR4RS principles (n = 21). 23% (n = 7) had not heard of FAIR4RS, and 7% (n = 2) were unsure.

Among the respondents who had heard of the FAIR4RS principles (n = 21), levels of familiarity varied. 33% (n = 7) reported being *extremely familiar* with FAIR4RS, and another 33% (n = 7) indicated they were *moderately familiar*. 19% (n = 4) said they were somewhat familiar, 10% (n = 2) were slightly familiar, and 5% (n = 1) were not at all familiar with the FAIR4RS principles.

### Relevance of FAIR4RS principles to research funding organizations

Respondents who reported being familiar with FAIR4RS principles were asked an open-ended question about the relevance of these principles to their organizations’ work. Most did not respond to this question. One respondent said it would be on the organization’s agenda soon: “[We] will be discussing in board about in this year.” Another respondent described FAIR4RS’s relevance in general terms:“Software underpins much of modern research and innovation, therefore I think that these are very relevant to [my organization]”. The two more detailed responses focused on (1) the benefits of FAIR4RS for science and (2) the limitations in the capacity to engage with it.

Here, similar scientific benefits of implementing FAIR and FAIR4RS are identified:

“As an organization supporting researchers, we value the FAIR and FAIR4RS principles as principles enabling more/better science. Following these principles and working with researchers willing to adhere to those provides us more chances to deliver better technical solution to their scientific computational challenges.”

However, another respondent’s enthusiasm for FAIR4RS was tempered by the limited bandwidth available to address this concept.

“The FAIR4RS principles are extremely relevant to my organization. However, […] any work we could do on FAIR4RS would have to be balanced against current priorities and capacity. One reason we haven’t done much on research software (or embedding FAIR4RS) is because we’ve got our hands full with other open science issues (e.g., open access publishing, data management and sharing, promoting FAIR data, etc.).”

This research funder response suggests an implicit prioritization within the range of recognized open science principles and practices, with articles and data coming before software.

## Discussion

There is near-universal awareness of FAIR data principles among our respondents, with 97% indicating they had heard of them. Moreover, a significant majority of these funder representatives reported high levels of familiarity, with 73% being ‘extremely familiar’ and the remaining 27% ‘moderately familiar.’ This widespread recognition underscores the successful dissemination and integration of the FAIR principles within international government research policies and research funding organizations. This shows a collective acknowledgment of FAIR data’s importance in advancing scientific progress.

In this study, we used awareness of FAIR data principles as a baseline against which to compare FAIR4RS awareness. This benchmark shows that awareness of the FAIR4RS principles for research software is substantial but notably lower than that of the FAIR data principles. 70% of respondents had heard of FAIR4RS, and familiarity levels were more varied: only 33% were ‘extremely familiar,’ another 33% ‘moderately familiar,’ and the rest less so. This disparity between FAIR and FAIR4RS awareness suggests that while the importance of research software is increasingly recognized, the specific principles guiding its management and sharing have not yet achieved the same level of penetration within the funding community as the data-focused FAIR principles. Beyond awareness, the open-ended responses signaled that implementing FAIR4RS within research software funding may be lagging behind awareness of the concept and principles.

This research fills a critical gap by providing empirical evidence on the awareness and adoption of FAIR and FAIR4RS principles among research funders. The insights gained can inform future efforts to develop targeted strategies for FAIR4RS promotion. However, given the uneven geographic representation in the sample, there is a need to expand this kind of research to explore the awareness and adoption of FAIR4RS principles in underrepresented regions and gain a more global perspective.

### Role of Funders in Advancing FAIR4RS Principles

Successful changes to the research system typically need to involve all relevant parties, albeit with varying levels of initiative and activity. Like using a lever to move a large boulder, there are different points at which systemic changes can be instigated. A key leverage point is with the funders themselves^19^. The research software funders we surveyed show that principles to improve research practices such as FAIR and FAIR4RS are on their radar. However, integrating such principles into funding programs and wider research policy can be daunting^20^. This challenge must be overcome to improve research software and science. Research funders play a pivotal role in shaping the research software community’s practices, and the study’s findings imply that funders could take several proactive steps. They could incorporate FAIR4RS into policies and funding requirements by embedding these principles into grant guidelines and evaluation criteria. This would encourage the adoption of good practices in software development and sharing. Additionally, funders could provide support and infrastructure by offering training, resources, and infrastructure support to help researchers and research software engineers meet FAIR4RS standards. Furthermore, funders could foster research software collaboration and community engagement by engaging with other funders, institutions, and organizations. Such collaboration can promote a collective effort to advance FAIR4RS principles, facilitating the sharing of good practices and developing joint strategies and monitoring and evaluation frameworks.

Additional relevant parties who have a potential role to play include policymakers, publishers, research performing organizations, professional societies, research infrastructure providers, and researchers themselves. Funders and policymakers often interact, though with different roles in different countries. Publishers similarly have a relationship with funding agencies, sometimes enforcing funder policies and, in other cases, facilitating the implementation of good practices developed by the research community. Professional associations are often key to developing and codifying such community-led good practices because research practices and norms vary by academic discipline. Research performing organizations can also contribute to such changes via hiring, promotion and compliance policies, training and organizational norms. Similarly, once practices such as FAIR4RS are being discussed and used by researchers, research infrastructure providers also begin to embed them into production systems.

The interaction between these different players in the research ecosystem can be unpredictable, so those trying to cultivate changes often aim to influence all relevant players to trigger a cumulative and cascading set of changes. Some level of success in one element of the research ecosystem can lead to other successes that are built upon in subsequent change initiatives. This iterative developmental process is happening with FAIR4RS via several initiatives targeting different aspects of the research ecosystem. ReSA and its Funders Forum are working with research funders through the ADORE.software declaration, the International Council of Research Software Engineering (RSE) Associations and its members are working with policymakers and research performing institutions, and the Research Data Alliance FAIR4RS working group and Software Source Code interest group are working with publishers. These and other initiatives will continue to drive the implementation of the FAIR4RS principles alongside a myriad of everyday small-scale, informal interactions that build momentum for change.

## Supplementary information

Human subjects checklist (completed)
