# Supplementary material for: Awareness of FAIR and FAIR4RS among international research software funders
Source: Sci Data. 2025 Apr 15;12:627. doi: 10.1038/s41597-025-04820-4 (PMC12000615; doi:10.1038/s41597-025-04820-4)
Supplement: Supplementary file 1 — Human subjects checklist (completed) [file 41597_2025_4820_MOESM1_ESM.docx]

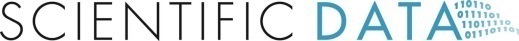
Human Data Submission Checklist

We request that all *Scientific Data* authors describing data that originated from humans (e.g. biological studies, medical data, personal information, survey responses, etc) supply information to the editorial office to support our data checking workflows.

**We kindly request you:**

- answer questions as directed (tick all answers that apply).
- confirm that the relevant information is also included in the main article file (see guidance notes after each question).
- sign the form (only one author is required).
- supply a copy within your manuscript files.

1. **Please tell us where the data came from:**

Authors of the study (you, other named authors) had direct responsibility for data collection from participants (e.g. a study, trial, survey).

- Please ensure the complete process for participant recruitment is described in the paper (e.g. advertising process, selection, screening, name of facility, etc)

Data was collected from patients via their routine medical treatment, diagnosis, consultation, or pathology assessment and then obtained from medical facilities retrospectively. Please also check this box for any other routine private data collection method performed outside of a study (e.g. a census, commercial origin, etc).

- Please ensure the origin of the data is clear in the paper, the name of the relevant institution/body holding the data is named, along with the date of collection and all other practical data collection details. Please also explain how the data was acquired by the you as the named author (e.g. a collaboration, data access request, etc).

Data was collected from Biobank samples.

- Please ensure the Biobank is named in the paper, plus any relevant sample numbers, and ensure all policies from the supplier have been followed. Re-publishing details of primary collection methods (i.e. how samples were initially collected by the Biobank) may not be required if these can be cited within a public document unless you feel they are required by data users.

Data derives from biological material of other origin (e.g. cell lines).

- Please ensure the name the product and supplier of any commercially acquired material is included within the Methods. Other material requires full experimental details on the original collection methods if not previously published (the first box – ‘primary data collection’ - may be more applicable if so), or a citation to the relevant paper/documentation if this has been previously disclosed.

Data was downloaded from a database or repository (secondary data)

- Name the repository/database and all relevant accession numbers to allow others to retrieve the same input data.

For repository/database data, please check this box to confirm that you have checked the licence, data usage agreement, or other relevant policy to ensure that you re-distribution are usage within this dataset is permitted within those terms.

Synthetic data – mimicking real human data, but not relating to any real person.

- Provide the source of any real (human-derived) training data if relevant. This does not need to be re-shared within the synthetic dataset as some as users may source it from the original database (provide a URL or dataset citation) but there should be some viable method for users to repeat the data creation process.

Derived from public documents or other materials.

- Ensure all relevant data sources are included in the paper (e.g. specific citations or URLs from where the information was extracted) and that you have permission to do this.

Other (please state):

1. **What does the data contain?**

Choose all that apply:

Contains direct identifiers (names, identifiable facial images, biometric data, genomic or transcriptomic data)

State types:

Contains 3 or more indirect identifiers. This is checked as indirect identifiers may still reduce the sampled group to a potentially identifiable cohort (e.g. locations, gender, religion, ethic group, other demographic data, etc)

State types:

Contains sensitive or protected fields (e.g. racial or ethnic origin, political opinions, beliefs, union membership, health/diagnosis data, psychological assessments, financial data, criminal convictions, etc). These do identify the individual but prevent a risk of sensitive data disclosure if they can be matched to them.

State types:

1. **Please tell us how/if consent was obtained from participants:**

Informed consent for participation and data sharing was obtained directly from participants.

Informed consent for participation and data sharing was obtained from a parent, guardian, or other responsible role, in cases where the individual cannot reasonably consent themselves.

Informed consent was obtained indirectly, at the time of routine collection, for possible research use in future. Please also tick this box for non-clinical examples, e.g. data collection within apps or other passive methods where users have agreed to data sharing via agreements with terms and conditions.

- For any of the three above cases, please ensure the relevant details are stated in the paper. Who consented and what was specifically was consented to. For cases where data was obtained from a previous study or other party, and the data is being re-used, please state and cite the source of the original consent and ensure you have permission to re-use the data in this new work.

Patients were not informed or did not provide consent for data sharing (e.g. during routine a medical interaction, non-medical case) but a third party has agreed this may be waived.

- Please state this in the paper, naming the body who provided the waiver (e.g. institutional or hospital ethics board).

Data originates from a third party (database, biobank, etc), with consent or waivers managed by the primary data collector.

- Please check and confirm consent was acquired by the primary data collector and state this in the manuscript. This may be cited if the information is present on a third-party document or webpage.

Data originates from social media or other public documents.

- Please note that we still require consent from participants for compilation, annotation, and/or re-use of their data in most cases, even if it has been freely contributed to a public platform, unless the platform informs the user of any potential use or re-distribution and obtained an explicit opt-in to do so. Researchers may also need to check any copyright claimed by the platform owner. Please ensure all details are checked and stated in the paper.

N/A (e.g. the data is synthetic)

Other (please state):

1. **Please tell us what ethics approval has been provided.**

Institutional ethics board or IRB (this should match at least one author institution associated with the paper) OR a third party or private IRB (if no such board exists within the authors’ institution(s)) approved the data collection study.

- Please provide the name of the relevant board, explain what they have approved (e.g. approval to conduct the study and share the data), and share the relevant case number, in the paper. If the board has issued a waiver of consent, please state in the manuscript text why this waiver was issued, as per Q3.

Ethics approval is not required (e.g. for secondary data use – this journal does not mandate approval for these studies on the basis that the approval to share sits with the original data provider, however this may be required by your institutional policy).

Other (please state, and add any relevant information in the manuscript):

1. **Please tell us what licence the data are available under**

This should be clear on the repository page as a minimum requirement. We also suggest more complex restrictions on usage are stated in a “Usage Notes” section at the end of the main paper. Copies of any Data Usage Agreements (DUAs) should be visible at the repository for potential users in time for final publication. Please share a copy of the DUA within the manuscript files if these are not available at the repository at the time of submission. Links or copies of standard CC licences do not need to be provided or lined in the paper.

CC0 or CC-BY (or equivalent)

CC-BY-SA (only supported for secondary data where the primary source data are -SA licenced)

A Data Usage Agreement (DUA)

Other (please state):

**Note:** We do not support the use of CC-BY-NC or ND licences as a means of managing personal (participant) data protection. Researchers requiring controls beyond CC0 or CC-BY are encouraged to use a formal Data Usage Agreement (DUA) stipulating what users can or can’t do with the data being shared. Please contact us prior to submission ([scientificdata@nature.com](mailto:scientificdata@nature.com)) if you require assistance with this.

1. **Please tell us what practical controls for data access are required.**

No controls (user download without login or registration)

- There is no requirement to state this explicitly in the paper if the repository interface is intuitive and users can easily discern how to download the data.

Automated user registration and verification without manual screening (e.g. users receive a download link or credential access immediately, providing a valid email address, name, agree to terms, etc)

Manual application or registration process via the repository (beyond basic email validation).

- For the above cases, please ensure any application criteria or complex processes are also stated in the Usage Notes section of the manuscript.

Other (please state):

1. **Please tell us how reviewers may access the data:**

Our requirements are for standard reviewer anonymity to be preserved and for access to be available within a reasonable reviewing timeframe (most reviewers are given 10 days to assess the paper and dataset)

Free download of open data (no access controls / anonymous download / reviewers can use the same process as any other user)

Registration required, but user credentials are not checked or visible to any of the authors listed in the paper.

- The above two examples do not need special explanation to manage peer review – reviewers will access the data via the same process as any normal user.

Registration required, but application process may be bypassed for peer reviewers (e.g. via a private link, or download)

Reviewers may check a small, representative sample of the data that may be shared to support peer review (where removal of access controls in not required but this is safe to do so), with a link shared in the paper

Other (please state):

- For all bespoke reviewer access method where reviewers need to bypass the standard data access process, please EITHER add temporary details in the manuscript (which will need to be removed for final publication) OR share a “Data Access Note” document within manuscript files as an “Article” type, appended to the front of the paper as the first item in a merged pdf. These should include full details for how reviewers may access the data, including links, passwords, etc and will be visible to all individuals invited to assess the paper. In all cases, the reviewers identity needs to be hidden from the authors and the process should facilitate data access within a reasonable timeframe.

I certify that all the above information is complete and correct.

28 January 2025

Eric A. Jensen

Typed signature Date

Please use additional pages if further details are required.
